# Postpartum Depression: Screening, Diagnosis, and Management Programs 2000 through 2010

**DOI:** 10.1155/2012/363964

**Published:** 2012-07-30

**Authors:** Barbara P. Yawn, Ardis L. Olson, Susan Bertram, Wilson Pace, Peter Wollan, Allen J. Dietrich

**Affiliations:** ^1^Department of Research, Olmsted Medical Center, Rochester, MN 55904, USA; ^2^Departments of Pediatrics and Community and Family Medicine, Dartmouth Medical School, Dartmouth Medical School, Hanover, NH 03755, USA; ^3^National Research Network, American Academy of Family Physicians, Leawood, KS 66211, USA; ^4^Department of Community and Family Medicine, Dartmouth Medical School, Hanover, NH 03735, USA

## Abstract

The value and appropriateness of universal postpartum depression (PPD) screening remains controversial in the United States. To date, several PPD screening programs have been introduced and a few have been evaluated. Among those programs that have been evaluated, most report screening rates, diagnosis rates, or treatment initiation rates. Only four studies included patient outcomes such as the level of depressive symptoms at 6 to 12 months postpartum, and only two reported success in improving outcomes. Program characteristics that appear to result in low rates of diagnosis and followup after PPD screening include requirements for a formal psychiatric evaluation, the need to refer women to another site for therapy, and failure to integrate the PPD screening into the care provided at the woman's or her child's medical home. The two programs that reported improved outcomes were both self-contained within primary care and included specific followup, management, and therapy procedures. Both resulted in the need for outside referrals in less than 10% of women diagnosed with postpartum depression. Future studies should be based on the successful programs and their identified facilitators while avoiding identified barriers. To affect policies, the future program must report maternal outcomes going beyond the often reported process outcomes of screening, referral, and therapy initiation rates.

## 1. Introduction

Postpartum depression (PPD) is common, reported to be experienced by 15% or more of women during the 12 months after they deliver [1–4]. Despite nearly universal health care encounters at some time during this period, only about 50% of women with significant depressive symptoms are recognized [4–6]. PPD occurs among women of all ages, parities, races, and socioeconomic groups [7, 8]. Untreated and unresolved PPD adversely affects the woman, her infant and her relationship with family members [9–12]. Therefore, universal screening for PPD is an attractive approach, and several groups have made recommendations regarding universal PPD screening in the maternity or early well child care setting [2, 13–19]. 

Many United States (US) as well as international programs have attempted to implement universal postpartum depression screening with or without followup care. Some programs have included formal evaluations, but few have resulted in improved patient outcomes [20–30]. Reviews by several evidence-based guideline groups have reported insufficient or inconclusive information regarding improved outcomes with PPD screening, preventing them from recommending universal PPD screening. While these reviews provide summary assessments, they fail to assess program design, context, setting, or components of the program as potential factors influencing success or failure [2–4, 15, 18, 25]. 

This paper also reviews the published evidence regarding PPD screening and follow up, describing the methods and outcomes reported for recent published PPD studies [20–24, 27–30]. Unlike the reviews completed for the guidelines panels, our goal is to summarize elements of program design, program setting, and program components as they may relate to program success in not only providing PPD screening but improving outcomes of women judged to have PPD.

## 2. Methods

We reviewed PPD screening programs described in the English language peer-reviewed literature between 1998 and 2011 [20–24, 27–32]. Studies were identified from Medline/PubMed, PsychINFO, and Cinahl using title, abstract and keyword searches for the terms “postpartum depression,” “maternal depression,” and “perinatal depression,” each independently and then linked to “screening.” We also identified work published in the same time frame from the references in the Institute of Medicine's (IOM's) report on parental depression [1]. Finally a Google search using the phrase “postpartum depression screening programs” was completed to identify reported puslihed in nonindexed health care and social science journals. Only programs that reported an intervention and some type of outcomes such as screening rates, rates of screen failure, diagnosis rates, or maternal depression outcomes were included. The programs described here vary from small studies done in three to seven clinics in a single city to regional, state, and national programs. The published reports of several US programs were supplemented by additional information from the first or senior author of the paper(s) describing the programs (personal communications Kim Yonkers, MD, Dwenda Gjerdigen, MD and Ardis Olson, MD).

## 3. Results

Overall, reports on 54 programs were identified but only eight of those had both clearly stated interventions and outcomes resulting in their inclusion in this report (Table 1). The total number of women included in these programs is difficult to ascertain since little data was provided on the number of potentially eligible women in some of the largest of the programs [22, 28–32]. All of the programs were designed to address postpartum women from less than a week after birth [20, 21] to 6 months postpartum [22]. Five programs were limited to patients literate in English. The Hong Kong program was limited to Chinese [26]. Two programs, the New Haven [22] and the family medicine program [27], allowed either English or Spanish. Characteristics of the women enrolled, such as insurance, marital and socioeconomic status (SES) were variably reported and when reported differed by program and country. In the US, Medicaid was the most common insurer and coverage often ended at 6 to 8 weeks postpartum [29]. The New Jersey program was open to all women, but only those with Medicaid insurance were included in the outcomes assessment [29]. In Australia and in Hong Kong, national health insurance assured that all enrolled women received insurance coverage for the entire prenatal and postpartum period [26, 28, 30].

## 4. Program Characteristics

### 4.1. Screening

All programs used the EPDS [21, 27, 33], the PHQ-9 [27, 34], or the PHQ-2 [35–38] for PPD screening. One program used a combination of the EPDS with all elevated scores being further assessed by the PHQ-9 [27, 39]. The cutoff scores for normal versus elevated were similar for all programs: an EPDS of 10 or greater or a PHQ-9 of 10 or greater [33, 34, 39]. 

Screening was most often reported to be initiated at the site of well child or maternal postpartum care [21, 23, 26–32]. The New Haven program was an exception. Designed as a community-based program, women were referred or self-referred to the community program office where they were screened and could be further evaluated [22]. Screening rates varied from a low of 33% to over 95% of eligible women in the US clinic-based programs [21, 27]. In Australia, the screening rate was estimated to be approximately 40% in one region of the country [31, 32]. The New Haven and New Jersey programs did not report screening rates [22, 29]. However, screening was assumed to be limited since participation in the physician educational offerings for these programs was low, with only 58% of obstetricians, 13% of pediatricians, and 12% of family physicians attending PPD education offered to support the New Jersey statewide program [29]. 

Procedures for further evaluation of elevated screening scores were not uniform across the programs and not all programs included plans for management or monitoring of women with diagnosed depression. For example, the screening programs in Australia, New Jersey, and Olmsted County, MN, US [23, 24, 28–32] did not report any specific follow-up procedures for further evaluation of elevated screening scores. In three programs procedures for evaluation of women with elevated screening scores required referral to an offsite mental health clinics or to delayed visits to staff at the screening site [20, 22, 33]. Those referrals were reported to include the Structured Clinical Interview for DSM Disorders (SCID) which is a formal interview that requires 30 to 45 minutes to complete and is usually administered by a psychologist or psychiatrist (http://www.SCID4.org/) [40]. Only two programs maintained screening and evaluation within the same site. Both of these programs also included specific procedures and support for PPD management and monitoring. Neither included evaluation with the SCID [26, 27]. 

## 5. Outcomes

 All programs except the New Haven program reported one or more process outcomes such as screening rates (see above). The Olmsted County, MN program reported increased rates of PPD diagnosis and treatment initiation following universal PPD screening [24]. The New Hampshire program, based in pediatricians' offices, reported “referral or maternal support from the pediatrician” in 67% of women with elevated screening scores. Buist et al. reported some increase in PPD diagnoses and therapy initiation in a subset of women screened in the Australian program [31, 32]. 

Four programs reported maternal outcome information [22, 26, 27, 29]. Both the New Haven Healthy Start and the New Jersey state programs reported no increases in any PPD-related clinical activities including no increases in rates of diagnoses or numbers of PPD related office visits [22, 29]. The Hong Kong and US primary care program reported both improved process outcomes and improved maternal outcomes at 6 or 12 months [26, 27]. Both reported declines in maternal depressive symptoms at either 6 or 12 months as well as high rates of screening, increased rates of PPD diagnosis and therapy initiation.

### 5.1. Reported Barriers to Success

 In the New Haven [22] and Minneapolis and St. Paul studies [21], evaluation of women with elevated screening scores required an in-person or telephone-based referral to a mental health professional for an SCID assessment. The requirement for an SCID assessment appeared to be a major barrier [40, 41] for completion of PPD evaluation with a low percentage of women in the Minneapolis/St. Paul study program completing the referral visit for this SCID assessment. A similar pattern of failure to participate in the SCID evaluation was seen among the women in New Haven even though they could do the interview by telephone (personal communication Kim Yonkers, MD, February 21, 2012).

### 5.2. Possible Facilitators of Success

 The Hong Kong and US family physician programs both introduced specific evaluation procedures to be completed with women with elevated screening scores and management and monitoring systems for those with diagnosed depression [26, 27]. In both cases, the procedures and care systems changes were based in the usual care site and usually began immediately after screening. To support the follow-up procedures and care system changes, the Hong Kong program provided a 12-hour educational experience for the nurses who completed screening in the well child visits. The nurses were taught some basics of supportive therapy and motivational interviewing [26]. In the US family medicine program short staff educational sessions (2 hours total), support tools for diagnosis, therapy selection and initiation, schedules of recommended management and monitoring visits, content outlines for nurse calls as well as an immediate action plan (IAP) to guide assessment of suicidal ideation [42] were provided to the practices. The PHQ-9 was used as a metric for assessing response to therapy and timing of remission in both the Hong Kong and US programs [26, 27]. Referral for complex problems occurred in about 6% of the women in each of the studies [26, 27]. 

## 6. Discussion

While the impact of PPD screening clearly differs among published studies, so do the programs that support that screening. To assess the reported results without considering the context in which screening was completed is likely to miss opportunities to identify parameters of successful translation of PPD screening into daily practice. It is important to recognize the complexity of the process of changing both clinician behavior and establishing effective office systems to consistently screen and intervene. In addition, the type of results reported must also be considered [43–47]. Screening rates, therapy initiation, or intent to treat are important preliminary results but must be followed by studies that assess and report patient outcomes [1, 12, 15, 16, 19]. Patient outcomes such as the level of depressive symptoms or rates of remission need to be assessed and reported in the context of the full program that achieved those outcomes [26, 27]. With a limited number of outcome studies published to date, the controversy surrounding the value of universal PPD screening continues.

Among the studies reviewed here, four programs reported screening at least two-thirds of eligible women and in all instances the programs were conducted within a woman's or her infant's usual care site [20, 23, 26, 27]. In these practices, PPD education was provided to the clinical and support staff within the practice site [20, 23, 26, 27]. In the largest programs, all of the state of New Jersey and all of Australia, plus the New Haven Healthy Start program, educational support for community physicians was not tailored to individual practices and only modestly attended. Providing education alone has been shown previously to not facilitate practice change [43–46, 48, 49]. 

Screening alone has not been shown to improve patient outcomes and may be unethical [13, 50]. Dealing with PPD is limited to adding ten questions to a patient care visit. Even a negative screening result requires some discussion. A positive screening result should trigger a cascade of events that change the visit content and practice work flow. Practice tools are required to facilitate these changes. Olson and her colleagues have developed a set of tools to facilitate the introduction and implementation of routine PPD and maternal depression screening into pediatric practices. The information is available on a website [51, 52] and has been widely disseminated through an American Academy of Pediatrics Task Force report [53]. Yawn et al. also developed primary care specific tools to support diagnosis, medication therapy initiation, and PPD followup and monitoring plan [27, 42]. Programs that support practice system changes such as universal PPD screening appear to benefit from local education and support for the practice change and its implementation. Without tools and support for system change, dissemination is likely to be slow with each practice reinventing similar work flow, education and support systems [45, 54]. 

Four studies did report patient outcomes, two without improvement (New Haven and New Jersey) [22, 29] and two with improved outcomes (Hong Kong and US family physicians) [26, 27]. Two important aspects that appear to differentiate successful and unsuccessful programs are the ability to provide the majority of care within the screening practice and the provision of education, tools and support for depression diagnosis and ongoing management to facilitate systems change. Primary care practices can provide care for major depressive disorder [47–49]. The Hong Kong and US family medicine studies suggest that this can include care of maternal depression [26, 27]. Referral to mental health specialists will continue to be necessary for the most complex patients and those who do not respond to primary care therapy. In both of the successful programs the referral rate to psychiatrists was 6% of screen positive women [26, 27]. However, with the worldwide shortage of mental health professionals [55] it is important that these resources be used for complex cases and that efforts are made to integrate mental health into primary care [42, 56, 57]. While some integration may mean bringing mental health professionals into primary care offices, an alternative may be to provide mental health services by the people already within the practice, the primary care physician supported by the primary care team [27, 43–45, 48, 49, 57, 58]. 

Requiring a SCID to diagnose PPD requires women to go outside their usual care sites and appears to be a major barrier to evaluation and diagnosis [21, 41]. Gjerdingen and others have similarly reported this potential barrier to PPD care [21, 57]. The need for the SCID assessment is based on results from studies of high-risk patients with complex mental health problems and who are referred to psychiatrists [1, 2]. These results may not be generalizable to a lower-risk primary care population being assessed within their continuity practices. The benefits of requiring a SCID for PPD diagnosis must be weighed against the risks of missing PPD and a chance to provide therapy and care to the women identified by screening without a SCID confirmation. Implementation of PPD care in primary care practices is feasible. Screening linked with appropriate and patient acceptable evaluation, monitoring, and depression management support can improve outcomes at 6 to 12 months postpartum. With education and support, primary care is likely to be able to provide the required evaluation and care for the majority of women with PPD leaving the limited numbers of mental health professionals to care for more complex cases. To date, the number of studies demonstrating positive patient outcomes is limited and additional studies and dissemination programs with careful evaluation will be required to confirm these results. In the future, primary care management of depression, including postpartum depression could be similar to primary care management of other chronic conditions such as hypertension, diabetes, and asthma where most patients can receive most of their care within the primary care setting and that care can meet the high standards of quality metrics [59]. 

## Figures and Tables

**Table 1:** 

Site	New Haven Healthy Start Program [22] (*n* = 1,336)	Minneapolis and St. Paul, MN, screening at well child programs [21] (*n* = 506)	Australia, universal PPD screening [28, 30–32] (*n* unknown)	US family medicine practices [27] (*n* = 1,263)	Olmsted County, MN universal PPD screening program [23] (*n* = 342)	New Hampshire, screening at all well child visits in pediatric practices [20, 33] (*n* = 1,398)	New Jersey State wide initiative [29] (30,955 analyzed)	Hong Kong Program [26] (*n* = 462)
Enrollment criteria	Low-income women within 6 months of delivery (i) self-referred (ii) referred by outreach worker	Women bringing children to 0-1 month to well child visits at family medicine or pediatric clinics	All postpartum women in all practices in the country	All women between 4 and 12 weeks PP coming for PP or well child visit to 28 enrolled practices	All women between 4 and 9 wks PP coming for PP visit to OB or FM to Olmsted County, MN provider	All women bringing children 0–18 years for well child visits for 6 month time period in three enrolled pediatric practices	All women with Medicaid insurance for during pregnancy and 1st yr PP were used in analyses, all women in state delivering an infant during time of interest were in program	All women visiting maternal and child health centers for 2 month well child check. Exclude if already receiving mental health care
	(English or Spanish)	(English only)	(English only)	(English or Spanish)	(English only)	(English only)	(Unknown)	(Mandarin only)

Site characteristics	New Haven Healthy Start initiative—a community-based program not within any clinic	Seven family clinics, 4 urban family medicine, and 3 suburban pediatric clinics	All clinics providing postpartum care in the country	28 US FM practices including rural, urban, and residency practices	All OB and FM postpartum care providers in the community	Rural Peds practices, all pediatric providers (pediatricians and nurse practitioners)	All maternity and well child practices in New Jersey	One maternal and child health clinic in Hong Kong, nurse run and staffed. Support from local psychiatrists
	Screening by staff	Screening by staff	Screening by staff	Screening by clinic staff and physician review	Screening by staff, review by physician	Screening by staff, review by clinicians	Screeners unknown	Screening by nurses

Screening tools	PHQ-9 (cutoff for followup 10), PTSD screener, anxiety and alcohol screener	PHQ-2 and then PHQ-9	EPDS	EPDS follow by PHQ-9 for all scores greater than 10 versus usual care with no formal screening	EPDS, score >9 considered high risk for PPD	PHQ-2, (scored 0–6) with cut point of 3 or more for positive screen	Left to the practice following an educational program to introduce screening and PPD management to physicians and other clinicians	EPDS, score >9 considered elevated compared to usual clinical assessment by nurse

Diagnostic methods	Telephone interview by master's level clinical social worker	Referral for SCID to mental health clinic	Unknown, primarily screening program	PHQ-9 and physician assessment	Physician or other clinician choice	Clinician discussed and offered referral resources	Physician or other clinician choice. Education covered both PPD care and referral	Onsite counseling by nurse trained with short program. Could go for additional referral

Followup program	Referral for therapy and calls by social worker at 1, 3, and 6 months for further referral suggestions Community education	As per mental health care professional to whom the patient is referred	Unknown	Detailed followup program and tools to support care, medication, and counseling use and schedule nurse calls	None provided	As per mental health professional to whom the patient is referred	Followup as determined by care providers	Followup was single session by trained nurse with optional additional counseling

Outcomes	Rates of therapy, levels of symptoms monthly after referral	Rates of screening completion including SCID	Rates of screening	Rates of screening, diagnosis, therapy initiation, levels of depressive symptoms at 6 and 12 months PP	Rates of PPD diagnosis and rates of PPD therapy initiated	Rates of screening completed and screen positive status, and rates of women willing to take action plus rates of pediatrician support offered	Rates of depression care initiation and continuation of care after 90 and 120 days	Levels of depressive symptoms at 6 and 18 months PP

Results	No change with program (before and after assessments)	Less than 33% completed screening and assessment with SCID	Rates of screening <40% in several regions	Increased rates of PPD diagnosis, therapy initiation, and lower levels of depressive symptoms at 12 months PP	Increased rates of PPD diagnosis and increased rates of PPD therapy initiated	Screening completed at 67% to 74% of well child visits. 6% of women had scores ≥3 Among screen + mothers: 47% thought might be depressed and willing to take action. 28% thought stressed, not PPD. Clinician action in 62%	No change in rates of care initiation or continuation (before and after the onset of the statewide program assessment)	Risk ratio of EPDS <10 was 0.50 for intervention versus usual care and NNS was 25 to prevent one EPDS of >10 at 12 months

Study design	Pre- and post-“open label”	RCT	Cohort	RCT	Pre- and post-cohort	Cohort	Pre- and post-study of Medicaid subset of population	RCT

Depression monitoring metrics	PHQ-9 score	Unknown	None	PHQ-9 score	None	N/A	None	None

Support systems and tools	Social worker phone calls, weekly drop in programs for behavioral health or pharmacological services, provided at no cost	None	None	IAP, medication table, nurse call scripts, self help tools, father's pamphlet, and monitoring schedule	None	N/A	Education attended by 38% of obstetrical care physicians and other clinicians and 16% of pediatricians and 12% of family physicians in New Jersey	Nurses doing counseling had 12 hours of training and could refer to psychiatrist if desired

Reported barriers to success	Adding mental health people into practice without integration may have decreased physician role as screener and evaluator, low SES population	Need to refer offsite for SCID	Unable to get EPDS screening integrated into many practices. A national program without incentives	Time barriers for clinic nurses to make calls, loss of insurance at 6 to 8 weeks PP for many of the women, failure to address PPD as chronic condition	No followup program included	Pediatrician role limited to screening, discussing impact on child, referral and short-term followup	Less than one-third of clinical care providers participated in the educational program	More than half of the women attending the clinic were ineligible including several who had already undergone PND screening
